# Impact of Organizational Support and Social Capital on University Faculties’ Working Performance

**DOI:** 10.3389/fpsyg.2020.571559

**Published:** 2020-10-23

**Authors:** Zhong Chen, Dong Chen, Michael Yao-Ping Peng, Qingchang Li, Yangyan Shi, Jialu Li

**Affiliations:** ^1^Research Center of Open Economics and Trade, Fuzhou University of International Studies and Trade, Fuzhou, China; ^2^School of Marxism, Minjiang University, Fuzhou, China; ^3^School of Economics & Management, Foshan University, Foshan, China; ^4^Marketing Department, School of Business Administration, Jimei University, Xiamen, China; ^5^Business School, Guilin University of Technology, Guilin, China

**Keywords:** higher education, institutional performance, social capital, resource-based view, organizational support

## Abstract

As the major concerns of higher education institutions (HEIs) are teaching, services, and research, this paper describes a region-wide evaluation of institutional performance in relation to universities in Taiwan. The evaluation was based on the perceptions of university professors regarding institutional slack and reputation, as well as internal and external social capital. The study sought answers to several research questions and adopted a survey approach. After choosing 30 universities of various sizes and from different regions, 926 professors were selected randomly as participants. Using PLS-SEM, this study confirms the influence of institutional slack, reputation, and internal and external social capital on performance. The results show that (a) institutional slack and reputation positively affect institutional performance; (b) internal social capital positively and significantly influences the relationships of institutional slack and reputation with performance; and (c) external social capital has a positive moderating effect on the relationship between institutional slack and performance. Concluding this paper, theoretical and managerial implications and suggestions for future studies are proposed.

## Introduction

As the people-to-people competitive mode extends to the state-to-state one (Marginson, 2007), globalized competition is becoming increasingly fierce and impacting the development of higher education, and in turn demographics and class sizes in higher education institutions (HEIs) (Altbach et al., 2009). Due to the ever-changing nature of higher education, a static view cannot be applied to the current environment (Findler et al., 2019). Specific to the changes seen in HEIs, it is important to ensure quality and fairness of teaching and learning (Maringe and Sing, 2014; Manatos et al., 2017; Veer Ramjeawon and Rowley, 2018).

In 2014, the stagnant economy decreased the total fertility rate in Taiwan to 1.11, compared with Japan’s 1.40, South Korea’s 1.25, and Singapore’s 0.80. Additionally, Taiwan’s college enrollment rate by entrance examination has been above 95% in recent years. Thus, Taiwanese HEIs have been approaching saturation regarding development intensity, so their education pattern has transformed from elite to mass education (Taylor et al., 2013). Particularly, after joining the World Trade Organization in 2001, Taiwan opened up its education market, which brought fiercer competition among its HEIs and thus new challenges (Shin and Harman, 2009). This caused an imbalance between supply and demand in the market. Therefore, exploring the development and performance of Taiwanese HEIs is an interesting research avenue and could provide generalizable results.

Literature on organizational management identifies keys to success that can be roughly divided into internal and external factors (Huang and Knight, 2017; Veer Ramjeawon and Rowley, 2018; Findler et al., 2019). Regarding internal factors, scholars have focused on the quantity and attributes of internal resources using the resource-based view (RBV), suggesting that the development and performance of an organization depend on its resources (Barney, 1991; Hitt et al., 2016; Huang and Knight, 2017). Furthermore, the corresponding measurement of resources is classified into reputation (Boyd et al., 2010; Plewa et al., 2016) and slack resources (SRs) (O’Shea et al., 2005; Voss et al., 2008; Su et al., 2009; Vanacker et al., 2017), which are essential factors within the RBV.

The internal-resource perspective neglects channels by which external resources and knowledge are acquired (Walter et al., 2006; Benbow and Lee, 2019). Due to the intangibility, ambiguity, and social embeddedness of knowledge and resources (Veer Ramjeawon and Rowley, 2018), external relations have become key in absorbing external resources (Leana and Pil, 2006; Huang and Knight, 2017). The relational perspective complements shortcomings of the RBV, and external relations contribute substantially to the performance of HEIs. Relational resources may be derived from HEIs both internally and externally (Adler and Kwon, 2002; Huang and Knight, 2017; Benbow and Lee, 2019; Diriye, 2019; Suseno et al., 2020). Therefore, studies on both internal and external factors should extend the theory and concept to multiple types of social capital.

Through government funding subsidies or industry–university cooperation, HEIs’ use of resources and knowledge can help improve their teaching, research, and service performance (Ryan, 2005; Dai and Kittilaksanawong, 2014; Veer Ramjeawon and Rowley, 2018; Findler et al., 2019). However, whether resource inputs can really achieve corresponding performance outcomes remains untested. Studies show that the added value of resource inputs to educational outcomes has shifted from educational efficiency to educational effectiveness (Powell et al., 2012; Lane and Johnstone, 2013; Pritchard et al., 2015), yet few have analyzed this overall relationship structure (Diriye, 2019). Thus, this study posits that a resource acquisition mechanism is necessary to increase the fit between resources and outcomes so the efficiency of resource inputs can be maximized (Veer Ramjeawon and Rowley, 2018; Diriye, 2019). The mechanism will utilize internal social capital (ISC) and external social capital (ESC) as a bridge. Therefore, this study proposes and analyzes the overall “resource inputs–social relationship mechanism–performance” relationship structure and verifies the moderating effect of social capital on institutional resources and performance.

This study contributes to existing research in three main ways. First, it investigates the phenomenon of resource allocation among a unique breed of Taiwanese HEIs. Second, it emphasizes the importance of two key institutional slack and reputation that HEIs leverage for superior institutional performance. By exploring HEIs in Taiwan, this study provides empirical evidence that the complementary perspective (moderating effect) of internal and external social capital positively strengthens the impacts of institutional resource on performance. Finally, and more specifically, the study adopts partial least squares structural equation modeling (PLS-SEM) to verify the well-established conceptual framework with resource-based and social capital perspectives in the HEI context.

## Literature Review

### Resource-Based View

In organizational studies, size is always deemed a significant variable. Some theories hold that large organizations have competitive advantages because of their greater quantity of SRs (Hitt et al., 2016). SRs can be used to realize the organization’s goals and ensure ideal performance through transfer or reallocation (George, 2005; Huang and Knight, 2017). Under the high uncertainty of educational policy transformation, the influence of slack on the performance of Taiwanese HEIs is especially important (Tan and Peng, 2003; Su et al., 2009).

When HEIs inspect their internal resources, they encounter limitations regarding cognition and structure (Hitt et al., 2016). The rarity and absorption attributes of SRs help explain the causes of organizational behaviors. Taking rarity and absorption as measurements, Voss et al. (2008) divided SRs into (1) financial slack; (2) operational slack; (3) customer relational slack; and (4) human resource slack. This study also applies this categorization to discuss the SR–HEI performance relationship.

According to the RBV, institutional slack can narrow internal boundaries, support innovation (Hitt et al., 2016; Vanacker et al., 2017), and help managers effectively respond to changing circumstances, thereby yielding competitive advantages (Huang and Knight, 2017). There are three reasons for discussing the positive relationship between institutional slack and performance in an open education market: (1) Most HEIs face a market environment with low stability. They highlight their unique characteristics to seize new opportunities and maintain their competitiveness, which means they support these innovative services or activities through unabsorbed SRs to enhance institutional performance (Vanacker et al., 2017). (2) Adapting strategic behaviors is important for HEI survival and development (Kraatz and Zajac, 2001); unabsorbed SRs can support existing strategic behaviors and dynamic adaptation to environmental changes, thereby improving institutional performance. (3) The tightness of educational resources makes it difficult for HEIs to obtain subsidies (Khanna and Palepu, 2000); thus, SRs may be valuable, unique, and inimitable and have strong implications for institutional performance.

Ryan (2005), discussing the relationships among financial resources, institutional expenditure, and student engagement from the perspective of HEIs’ teaching and service performance, showed that only instructional expenditure is significantly affected. This was also found by Dai and Kittilaksanawong (2014). Specifically, in people-oriented HEIs, slack faculty resources impact institutional performance, improving teaching quality (Manatos et al., 2017), academic performance, and service contribution. Thus:

H1: Institutional SRs have a positive relationship with institutional performance (teaching, research, and services).

The RBV regards reputation as an intangible asset comprising internal investment and external evaluation; thus, reputation can be defined as a series of general organizational characteristics (Roberts and Dowling, 2002; Huang and Knight, 2017). The value generated from relationships between these characteristics will develop into competitive advantages, generating performance advantages (Barney, 1991; Boyd et al., 2010; Hitt et al., 2016). Specifically, reputation can lower uncertainty through the transfer of valuable information. Studies on educational institutions have defined reputation according to (1) social cognition such as knowledge, impressions, and feelings and (2) social cognition depending on the minds of external observers (Rindova et al., 2005; Boyd et al., 2010; Plewa et al., 2016; Veer Ramjeawon and Rowley, 2018).

Institutional reputation is a broad construct comprising multiple elements, including quality and reputation (Plewa et al., 2016; Manatos et al., 2017). Scholars have proposed research structures at the university (Volkwein and Sweitzer, 2006) and college (Sweitzer and Volkwein, 2009) level and have verified the effect of reputation. The results show that (1) reputation is positively associated with enrolment scale; (2) reputation has a positive correlation with enrolment test scores; and (3) reputation positively impacts research performance (Plewa et al., 2016). Furthermore, high institutional reputation means high student quality (Plewa et al., 2016), so that effectiveness in both teacher engagement and student learning is achieved (Lynch and Baines, 2004; Siebert and Martin, 2013). Additionally, HEIs with high reputations are likely to collaborate with external academic institutions on academic research, sharing research experiences and academic information (Henry and Neville, 2004; Bergh et al., 2010). Therefore:

H2: Institutional reputation is positively associated with institutional performance (teaching, research, and services).

### Social Capital

Nahapiet and Ghoshal (1998) regarded social capital as a kind of organizational resource and defined it as current or potential embedded resources obtained by individuals or social units or transferred from social relationships (Huang and Knight, 2017; Diriye, 2019). Advantageous positioning and relationships within a network bring about abundant relational rents and competitive advantages for organizations (Dyer and Singh, 1998; Yli-Renko et al., 2002; Diriye, 2019). Kharouf et al. (2014) indicated that enhancing students’ trust in HEIs increases student satisfaction and retention and stimulates effective word-of-mouth descriptions of their respective HEIs. Drawing from Adler and Kwon (2002) and Leana and Pil (2006), this study explores the social capital–HEI performance relationship and verifies results from an integrated viewpoint. Here, social capital can be divided into ISC and ESC.

ISC can be defined as structural and relational content between individuals within the organization (Adler and Kwon, 2002; Benbow and Lee, 2019). Nahapiet and Ghoshal (1998) proposed that social capital comprises structural, relational, and cognitive dimensions. The structural dimension presents all patterns of ties between network members and explores their positions in the relationship network (Benbow and Lee, 2019). Information sharing encourages individuals to learn in a context of profound meaning (Leana and Pil, 2006; Huang and Knight, 2017). In the relational dimension, relationships are accumulated via a long-term interactive process (Nahapiet and Ghoshal, 1998) wherein behavioral norms develop between members through mutual trust, shared values, and interpersonal recognition (Leana and Van Buren, 1999; Benbow and Lee, 2019). Specifically, trust is key in establishing social networks, meaning that organizations should trust that members have the ability and willingness to exchange or combine knowledge and should lower the risk inherent in knowledge exchange (Leana and Pil, 2006; Kharouf et al., 2014; Veer Ramjeawon and Rowley, 2018). The cognitive dimension involves the common expressions, interpretations, and implications of social members, which can unify individuals’ behaviors (Leana and Van Buren, 1999; Diriye, 2019).

HEIs are committed to the derivation, integration, and transmission of expertise (Smylie and Hart, 1999; McLaughlin and Talbert, 2001; Diriye, 2019). The institutional reputation–performance relationship is affected by ISC because a high degree of ISC represents organizational cohesiveness. When organizational members are more united, their exchanges will be more frequent, as reflected in the views and identification of these members with their HEIs, transforming into positive cognition and awareness (Diriye, 2019). This can raise the subsidies provided by government departments or industries or strengthen industry–university cooperation. Moreover, information sharing among lecturers can help to improve teaching and research productivity. Combined with effective teaching methods, taking improved student learning outcomes as the objective (Smylie and Hart, 1999; Bryk and Schneider, 2002; Benbow and Lee, 2019), institutions can generate excellent word of mouth, thereby enhancing the positive impact of institutional reputation on performance, their responsiveness to changes in the external environment, and the creativity of internal knowledge (Adler and Kwon, 2002; Veer Ramjeawon and Rowley, 2018). Therefore:

H3a: The ISC of HEIs has a moderating effect on the relationship between institutional slack and performance.

H3b: The ISC of HEIs has a moderating effect on the relationship between institutional reputation and performance.

ESC mainly relates to the repeated connections (resources, relationships, and information) between a group of actors (individuals, groups, and organizations); it also explores why actors conduct specific interactions in different environments, what results will be produced, and actors’ positions in the relationship network (Laursen et al., 2012). With the opening up of Taiwan’s education market, the educational environment became harder to predict and control. In addition to exploiting existing resources and capabilities more effectively, HEIs should establish partnerships with other organizations to obtain more knowledge and abilities, overcome environment-related challenges, and maintain competitiveness (Adler and Kwon, 2002; Leana and Pil, 2006; Diriye, 2019). Therefore, establishing ESC enables institutions to share risks and new technologies, compete in the market, and supplement each other with economies of scale and technological advantages.

ESC has a moderating effect on the resources–performance relationship within HEIs. Compared with for-profit institutions, HEIs face intense public supervision and must manage multiple external relations. This includes the monitoring of social changes and transmission of institutional messages to related parties to enable them to understand and agree with management processes. Brammer and Pavelin (2006) argued that reputation is closely related to social responsibility. The performance of social responsibility means the establishment and maintenance of ESC, and HEIs must maintain close interactions with external related parties and solve diverse social issues, thereby enhancing their institutional reputations.

Additionally, during the process of ESC strengthening the institutional reputation effect, the person in charge of the university or college plays a key role in mobilizing external resources and government support while strengthening the institutional reputation (Smylie and Hart, 1999). That is, when the flow of educational subsidy sources is intervened by the government to limit the resource allocation, the relationship between HEIs and the government and industry will become a source of important resources (Luo, 2003; Benbow and Lee, 2019). Studies have proven that a good management system between institutions and government agencies helps institutions to obtain financial support, information flow, technology transfers, and other support to improve instructional equipment and management systems (Li and Zhang, 2007; Barasa et al., 2017), thereby enhancing teaching, research, and service performance and thus improving overall institutional performance. Thus:

H4a: The ESC of HEIs has a moderating effect on the relationship between institutional slack and performance.

H4b: The ESC of HEIs has a moderating effect on the relationship between institutional reputation and institutional performance.

**FIGURE 1:**
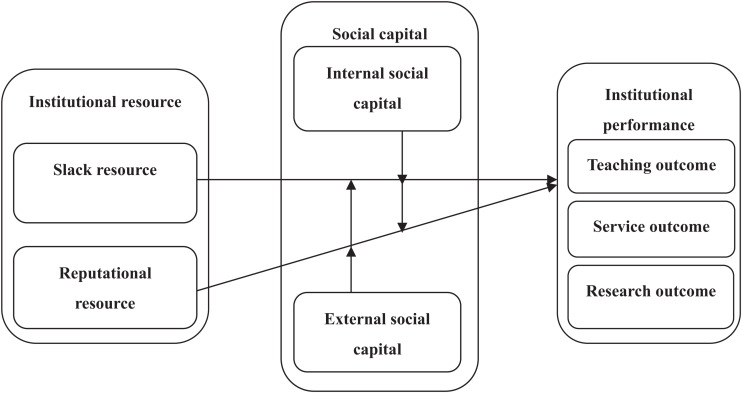
Research framework.

Based on the above descriptions, the following hypotheses are proposed in the Figure 1.

## Methodology

### Sampling

The research was conducted in Taiwan and employed stratified random sampling to collect survey data from full-time faculty members of Taiwanese HEIs. The database was taken from the annual list published by the website of MOE, which collects information on Taiwanese HEIs. The survey packages were posted to 2,000 faculty members of 30 HEIs in 2014. Each package contained a covering letter explaining the survey purpose, a survey instrument, and a pre-paid envelope. A total of 926 valid survey instruments were returned, giving an effective response rate of 46.3%. Regarding demographics, 66.8% of the respondents were male; 20.4% of the respondents were aged below 40, 22.8% were 41–45, 20.0% were 46–50, and 36.8% were over 51; 24.6% were professors, 34.6% were associate professors, and 40.8% were assistant professors.

The names of constructs are hidden in this study, and the question items are assigned randomly for common method bias (CMB) prevention. The Harman one-factor analysis method is used to test for CMB. The explained variance in one factor is 43.72%, which is smaller than the recommended threshold of 50%. Therefore, CMB was not problematic in this study (Harman, 1976).

### Measurement

Due to differences in countries’ educational policies, performance evaluations of HEIs vary. This study adopted measures from Marks and Printy (2003) and Douglas and Douglas (2006) and assumed that HEI performance can be assessed by examining teaching (five items), research (four items), and service outcomes (five items). Following Voss et al. (2008) and Su et al. (2009), this study held that SRs can be measured using financial, customer (student) relational, operational, and human resources; HEIs have been placed in the context of the research for the six-item ISR scale. Reputation was measured using Chaudhuri’s (2002) four-item scale and the “performance of graduates.” Based on Nahapiet and Ghoshal (1998); Yli-Renko et al. (2002), and Leana and Pil (2006), this study assumed that ISC comprises structural, relational, and cognitive dimensions. These were operationalized as information sharing, trust, and shared vision among faculty staff (six items each). To measure ESC, survey items were drawn from Laursen et al.’s (2012) two variables (social interaction and political participation) and measured using seven and three items, respectively. All scales are shown in Appendix 1.

### Data Analysis Strategy

The hypotheses of the research framework are tested, and paths are included in this study via structural equation modeling. For higher-order constructs (internal social capital, external social capital, and institutional performance), we reduced the number of parameters which are to be estimated following the partial aggregation method. This procedure involves averaging the responses of subsets of items measuring a construct. As internal social capital and external social capital are multidimensional constructs, we averaged responses of each dimension to serve as indicators for these constructs. Construct validity analysis was performed using the IBM-AMOS statistical program, v. 23.0, for Windows. Partial least squares structural equation modeling (PLS-SEM) was adopted to construct the structural model; specifically, verification of the structural model was performed using SmartPLS 3.0 (path analysis).

## Results and Analysis

### Reliability and Validity

All scales used were found to be reliable, with Cronbach’s α ranging from 0.77 to 0.91 (Table 1). Confirmatory factor analysis was employed to verify the scales’ construct validity (both convergent and discriminant). Hair et al. (2006) recommended convergent validity criteria as follows: (1) standardized factor loading > 0.7; (2) average variance extracted (AVE) > 0.5; and (3) composite reliability > 0.7. The evaluation standard for discriminant validity is the square root of the AVE for one dimension greater than the correlation coefficient with any other dimension. The standardized loadings ranged from 0.65 to 0.83, and most exceeded the 0.70 threshold. As Table 1 indicates, all three criteria for convergent validity were met, and the correlation coefficients were all less than the square root of the AVE, suggesting that each dimension had good discriminant validity.

**TABLE 1:** Assessing the convergent validity and discriminant validity of constructs.

Variables	1	2	3	4	5	6	7	8	9	10
1. Slack	*(0.74)*									
2. Reputation	0.59**	*(0.81)*								
3. Information sharing	0.62**	0.54**	*(0.76)*							
4. Trust	0.61**	0.61**	0.82**	*(0.81)*						
5. Shared vision	0.63**	0.60**	0.77**	0.82**	*(0.84)*					
6. Social interaction	0.62**	0.56**	0.61**	0.62**	0.64**	*(0.78)*				
7. Political participation	0.50**	0.45**	0.52**	0.50**	0.54**	0.67**	*(0.78)*			
8. Teaching outcomes	0.63**	0.58**	0.66**	0.66**	0.69**	0.60**	0.51**	*(0.81)*		
9. Service outcomes	0.70**	0.61**	0.68**	0.70**	0.69**	0.67**	0.56**	0.74**	*(0.71)*	
10. Research outcomes	0.55**	0.52**	0.54**	0.56**	0.57**	0.53**	0.39**	0.61**	0.69**	*(0.85)*
Mean	3.67	3.92	3.76	3.89	3.87	3.67	3.72	3.90	3.70	3.69
SD	0.68	0.70	0.62	0.63	0.65	0.62	0.65	0.58	0.62	0.73
α	0.87	0.90	0.89	0.85	0.93	0.91	0.81	0.91	0.83	0.91
CR	0.88	0.91	0.89	0.90	0.93	0.91	0.81	0.91	0.83	0.91
AVE	0.55	0.66	0.58	0.65	0.70	0.60	0.60	0.66	0.50	0.72

**p < 0.001. Diagonal (italic) elements are square roots of the AVE; note that AVE is not applicable for single-item measures.

### Test of the Structural Model

To test our hypotheses, we employed structural equation modeling (SEM) via SmartPLS. SmartPLS is a variance-based multivariate statistical program that is flexible when it is not possible to fit the strong assumptions of conventional covariance based on statistical programs (Vinzi et al., 2010). Furthermore, PLS accepts small sample sizes and can deal with complex causal models, which does not require multivariate normality and produces consistent parameter estimates (Nielsen and Gudergan, 2012). To assess the structural model, Hair et al. (2017) suggested looking at the R^2^, beta (β), and the corresponding *t*-values via a bootstrapping procedure with a resample of 5,000. They also suggested that, in addition to these basic measures, researchers should also report the predictive relevance (Q^2^) as well as the effect sizes (f^2^). As asserted by Sullivan and Feinn (2012), while a *p*-value can inform the reader whether an effect exists, it will not reveal the size of the effect. In reporting and interpreting studies, both the substantive significance (effect size) and statistical significance (p-value) are essential results which are to be reported (p. 279). Prior to hypothesis testing, the values of the variance inflation factor (VIF) were determined. The VIF values were less than 5, ranging from 1.762 to 2.495. Thus, there were no multicollinearity problems among the predictor latent variables (Hair et al., 2017).

**FIGURE 2:**
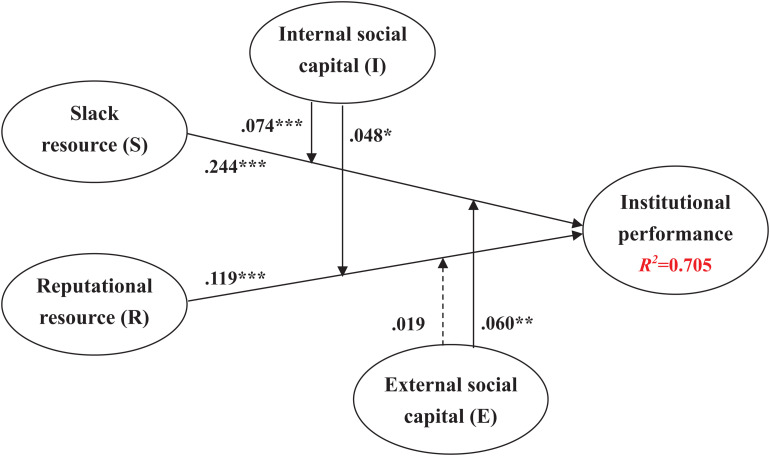
Structural model. ^∗^*p* < 0.05; **p < 0.01; ***p < 0.001.

The results of the hypothesized relationships and standardized coefficients are shown in Figure 2 and Table 2, including their respective standard errors and *t*-values. The results show that institutional slack (β = 0.244, *p* < 0.001) and institutional reputation (β = 0.119, *p* < 0.001) have a significant positive impact on institutional performance. That is, having more unabsorbed SRs within HEIs is more beneficial for enhancing resource utilization and improving the effectiveness of institutional members (i.e., teachers) in teaching, research, and services. Therefore, H1 is verified. Moreover, a good institutional reputation can be regarded as an important intangible asset of HEIs, not only showing positive recognition by external related parties but also enhancing the institution’s performance. Thus, H2 is verified.

**TABLE 2:** Results of the paths.

Paths	Std. β	*t*-value	Significance CI (2.50–97.5%)	VIF	*f*^2^
Institutional slack→ performance	0.244***	8.714	CI (0.191–0.300)	1.762	0.085
Institutional reputation→ performance	0.119***	4.075	CI (0.058–0.173)	1.820	0.022
I * S → performance	0.074***	3.325	CI (0.033–0.117)	2.382	0.011
I * R → performance	0.048*	2.769	CI (0.038–0.060)	2.214	0.011
E * S→ performance	0.060**	3.221	CI (0.048–0.074)	2.484	0.002
E * R → performance	0.019	0.616	CI (0.012–0.043)	2.495	0.002

*p < 0.05; **p < 0.01; ***p < 0.001.

This study also proposed that social capital affects the relationship between institutional resources and performance, aiming to verify the moderating effects of ISC and ESC. The empirical results in Figure 2 and Table 2 show that the moderating effects of ISC (β = 0.074, *p* < 0.001; β = 0.048, *p* < 0.05) have a positive and significant impact on relationship among institutional slack, institutional reputation, and institutional performance, representing that institutional performance is influenced by internal traits. If HEIs can accumulate moderate institutional slack and an irreplaceable institutional reputation, they can maintain competitive advantages. That is, by virtue of their ISC, HEIs not only promote the coordination and sharing of resources but also enhance effective management learning through long-lasting and repetitive interactions. Therefore, H3a and H3b are verified.

Finally, this study verified the effects of ESC on institutional resources and institutional performance. Figure 2 and Table 2 also show that the moderating effect of ESC (β = 0.060, *p* < 0.01) has a positive and significant impact on the relationship between institutional slack and institutional performance. This means that the formal and informal interactions and cooperation of HEIs with others create a free exchange of resources, reduce the cost of acquiring resources, and improve the reliability of identifying resources, which help the accumulation and integration of SRs and improve HEIs’ performance in teaching, research, and services. Therefore, H4a is verified. Nonetheless, the moderating effect of ESC (β = 0.019, *p* > 0.5) has no significant effect on the relationship between institutional reputation and institutional performance, so H4b is not verified.

## Discussion

Maintaining and promoting institutional performance are becoming increasingly important. Combining the RBV and the social capital perspective, this study explored factors affecting the performance of HEIs in Taiwan and the impacts of internal and ESC, institutional slack, and institutional reputation on institutional performance to provide new insights. Put another way, this study explored the relationship between resource inputs and performance outcomes from the perspective of social behavior, established a relatively complete relational structure, and verified its theoretical assumptions and relationships in a scientific way, thereby enhancing the richness of relevant theories.

The PLS-SEM results supported that institutional slack and reputation have positive correlations with institutional performance. That is, having a greater amount of SRs that are valuable, unique, and inimitable can help HEIs strengthen their strategic behaviors to cater to the dynamic and competitive higher education environment, thereby improving their teaching, research, and service outcomes. The results are similar to that of claims stated by Hitt et al. (2016) and Huang et al. (2017), resource-based view facilitate to provide more insightful explanations for improvement of institutional performance in higher education. Lawson (2001) highlighted the importance of SRs for institutions, including institutional funding, land ownership, government grants and subsidies, faculty, and other easily reconfigurable assets (Voss et al., 2008; Su et al., 2009). By integrating and allocating these resources, HEIs can create more knowledge advantage.

Furthermore, institutional reputation and slack are time dependent, and HEIs differ in this regard. As results of structural model showed, the path coefficient of institutional slack → performance is higher than the path coefficient of institutional reputation → performance. For example, the reputation of a public university is higher than that of a private university, which means that the latter takes more time to accumulate the same reputation. Thus, institutional slack has a greater impact on institutional performance than reputation does. Similar to findings by Tan and Peng (2003), this study verified that in a fiercely competitive market, HEIs have tangible resources that can be physically dispatched and utilized, and such resources are more important than intangible resources for performance.

Additionally, this study found that the moderating effect of ISC on the relationship between institutional slack and performance is positive and significant. This means that the trust and communication mechanisms among the internal faculties of HEIs improve the effectiveness and learning outcomes of internal actions in HEIs and promote intensive and in-depth social interactions between members (Tsai and Ghoshal, 1998; Persson, 2006), enhancing the efficiency of information, knowledge, and other resource exchanges (Auh and Menguc, 2005). Suseno and Ratten (2007) reached the same conclusion. With regard to the moderating effect of ISC, the results are consistent with the findings of Suseno et al. (2020), that is, provides support and evidence that institutional performance is facilitated through the leverage of social capital.

The moderating effect of ISC on the institutional reputation–performance relationship is also positively significant, which means that the more ISC an HEI has, the greater the impact of reputation on performance. As argued by Boyd et al. (2010), under the RBV, if institutional reputation is subject to both internal and external factors, HEIs will be able to use these to enhance their competitive advantages (Roberts and Dowling, 2002). Thus, institutional reputation can be considered a unique, valuable, and inimitable factor (Boyd et al., 2010) for superior institutional performance.

ESC also has a positive and significant moderating effect on the relationship between institutional slack and performance. Effective ESC is featured with the non-repetitive contact, resulting in complementary and non-overlapping resources. If exclusive SRs are consistently accumulated, HEIs will improve their performance to a high level. Therefore, the higher ESC with political attributes, the easier it is for HEIs to obtain tangible or intangible resources from government departments, such as financial aid, information, and research projects (Barasa et al., 2017). As Ryu (2017) claimed, by putting more time and effort into managing various aspects of social capital may be positively associated with managing institutional slack resource and improving institutional performance.

### Practical Implication

Based on the results, the following practical implications are proposed for how HEIs can enhance their institutional performance. First, managers of HEIs, such as principals, should prudently examine the resources owned, such as fund surpluses, full- and part-time lecturers, land, and buildings, and allocate and apply these in the most effective way. Second, to broaden the differentiation among universities, HEIs must emphasize their prominence in the market. HEIs can also gain positive social impressions and enhance their reputations through public welfare activities, thereby attracting more outstanding scholars to participate in academic cooperation, more enterprise companies to provide student internships and employment opportunities, and more industry–university cooperation opportunities. Third, HEIs should shorten their communication hierarchies and establish comprehensive quality-management cultures to enhance their administrative effectiveness (Manatos et al., 2017). Additionally, management could encourage faculty members to access diverse sources of information through group discussions, sharing of teaching cases, observations, and studies to promote the absorption and utilization of new knowledge. Finally, management should conduct in-depth cost–benefit studies through IR to improve the allocation and use of resources, subsequently enhancing performance indicators including academic leadership, resource allocation, teaching quality, campus atmosphere, and the maintenance of internal and external relations. Meanwhile, benchmark indicators should be used to compete with universities of the same type and understand the competition.

### Limitations and Future Directions

Although our study is significant for institutional and educational research, there are several limitations. First, it emphasized that relationships are a kind of dynamic resource with great mobility, however, the nature of dynamic resources will lead to different stages of relationship development, which can impact the accumulation of social capital. Therefore, as this study only used cross-sectional resources, it may not be generalizable from the viewpoint of dynamic relationship development. Thus, future studies could focus on the coevolution of social capital development stages and institutional performance.

Second, to further understand the organizational management of HEIs, future research could analyze the improvement and measurement of institutional performance under different theoretical perspectives, such as knowledge management, dynamic capability, and institutional economics. Finally, this study used HEIs in Taiwan as the research object, but different higher-education development policies will lead to differences in organizational structures, systems, and resource allocation. Therefore, future research could incorporate the performance of HEIs in different regions or countries for comparison.

Finally, the questionnaire distribution of this study was conducted in the years 2014 to 2015, and there were 926 valid pieces of questionnaire acquired, which may be time limited. However, changes of institutions of higher education in Taiwan are relatively stable, and there are still views and opinions which are consistent with the cognition of various indicators. Nevertheless, in order to improve the generalizability of the study, it is suggested that future studies may modify the relevant variables and conduct new investigations and further compare the differences among the study results, so as to provide new insights for the relevant theories and practices of higher education governance.

## Data Availability Statement

The raw data supporting the conclusions of this article will be made available by the authors, without undue reservation, to any qualified researcher.

## Ethics Statement

The studies involving human participants were reviewed and approved by the Institutional Review Board, University of Taipei. The patients/participants provided their written informed consent to participate in this study. The patients/participants provided their written informed consent to participate in this study.

## Author Contributions

This study is a joint work of all the authors. ZC and MP contributed to the ideas of educational research, collection of data, and empirical analysis. MP, ZC, QL, DC, and YS contributed to the data analysis, design of research methods, and tables. MP, DC, QL, and YS participated in developing a research design, writing, and interpreting the analysis. All authors contributed to the literature review and conclusions.

## Conflict of Interest

The authors declare that the research was conducted in the absence of any commercial or financial relationships that could be construed as a potential conflict of interest.

## Appendix

**TABLE 3:**

Construct	Variables	Items
Institutional slack	Institutional slack	Sufficient funds for teaching activities provided by our university.
		Sufficient funds for research activities provided by our university.
		Sufficient teaching facilities owned by our university.
		Sufficient human resources owned by our university.
		Sufficient research resources owned by our university.
		Good student satisfaction owned by our university.
Institutional reputation	Institutional reputation	Good reputation owned by our university.
		Our university is well-known.
		Our university is popular.
		High esteem owned by our university.
Internal social capital	Information sharing	Faculties engage in open and honest communication with one another.
		Faculties at this university have no hidden agendas or issues.
		Faculties share and accept constructive criticisms without making it personal.
		Faculties discuss personal issues if they affect job performance.
		Faculties willingly share information with one another.
		Faculties at this school keep each other informed at all times.
	Trust	I can rely on the Faculties I work with in this university.
		Faculties in this university are usually considerate of one another’s feelings.
		Faculties have confidence in one another in this university.
		Faculties in this school show a great deal of integrity.
		There is “team spirit” among Faculties in this university
		Overall, faculties at this university are trustworthy.
	Shared Vision	Faculties share the same ambitions and vision for the university.
		Faculties enthusiastically pursue collective goals and mission.
		There is a commonality of purpose among Faculties at this university.
		Faculties at this university are committed to the goals of the university.
		Faculties view themselves as partners in charting the university direction.
		Everyone is in total agreement on our university’s vision.
External social capital	Social interaction	Participation in cultural associations
		Participation in voluntary associations
		Participation in non-voluntary organizations
		Participation in various voluntary associations per region
		Meeting friends regularly
		Social meetings
		Satisfaction as to relationships with friends
	Political participation	Unpaid work for political parties
		Money given to parties
		Participation in political meetings
Institutional performance	Teaching outcomes	Teaching quality
		Instructional interaction
		Teaching creativity
		Teaching materials
		Course setting and arrangement
	Research outcomes	Publication
		Patent and innovative productions
		Plans and study projects
		University-industry collaborations
	Service outcomes	Internal operational procedures and fundraising within the HEI
		Students’ extra-curricular learning/academic assistance
		Instructing students to participate in various competitions
		Social service
		Public-benefit activities
